# Comparison of non-parametric methods for ungrouping coarsely aggregated data

**DOI:** 10.1186/s12874-016-0157-8

**Published:** 2016-05-23

**Authors:** Silvia Rizzi, Mikael Thinggaard, Gerda Engholm, Niels Christensen, Tom Børge Johannesen, James W. Vaupel, Rune Lindahl-Jacobsen

**Affiliations:** Max Planck Odense Center on the Biodemography of Aging, J.B. Winsløws Vej 9, Odense, 5000 Denmark; Unit of Epidemiology, Biostatistics and Biodemography, University of Southern Denmark, J.B. Winsløws Vej 9, Odense, 5000 Denmark; Danish Cancer Society, Strandboulevarden 49, Copenhagen, 2100 Denmark; The Norwegian Cancer Registry, PB 5313 Majorstuen, Oslo, 0304 Norway; Max Planck Institute for Demographic Research, Konrad-Zuse Str. 1, Rostock, 18057 Germany

**Keywords:** Aggregated count data, Ungrouping methods, Smoothing

## Abstract

**Background:**

Histograms are a common tool to estimate densities non-parametrically. They are extensively encountered in health sciences to summarize data in a compact format. Examples are age-specific distributions of death or onset of diseases grouped in 5-years age classes with an open-ended age group at the highest ages. When histogram intervals are too coarse, information is lost and comparison between histograms with different boundaries is arduous. In these cases it is useful to estimate detailed distributions from grouped data.

**Methods:**

From an extensive literature search we identify five methods for ungrouping count data. We compare the performance of two spline interpolation methods, two kernel density estimators and a penalized composite link model first via a simulation study and then with empirical data obtained from the NORDCAN Database. All methods analyzed can be used to estimate differently shaped distributions; can handle unequal interval length; and allow stretches of 0 counts.

**Results:**

The methods show similar performance when the grouping scheme is relatively narrow, i.e. 5-years age classes. With coarser age intervals, i.e. in the presence of open-ended age groups, the penalized composite link model performs the best.

**Conclusion:**

We give an overview and test different methods to estimate detailed distributions from grouped count data. Health researchers can benefit from these versatile methods, which are ready for use in the statistical software R. We recommend using the penalized composite link model when data are grouped in wide age classes.

**Electronic supplementary material:**

The online version of this article (doi:10.1186/s12874-016-0157-8) contains supplementary material, which is available to authorized users.

## Background

Histograms are the simplest and most used non-parametric density estimators. Depending on interval lengths and end points, the same data can reveal different shapes when coerced into histograms. This is particularly the case when histograms present wide and varying intervals, whereby information on the underlying pattern is lost. In epidemiology data are often available in such aggregated form. A typical case is age-specific death distributions where death counts are collected in age groups, usually of 5-years in length with an open-ended interval for the ages older than 85 years [1, 2]. With aggregated data at hand there is often the need to estimate age-specific distributions on a more detailed grid of ages, e.g. by single year of age, to compare, for example, nonagenarians and centenarians over time. This problem has been addressed in the literature and several solutions have been suggested for ungrouping age-at-death distributions [3] and fertility patterns [4].

The most widely used non-parametric methods to estimate distributions from grouped data are kernel density estimators and spline interpolations. A challenge is to efficiently ungroup data available in very wide intervals, e.g. age-specific death counts observed for the ages greater than 85 years, with non-parametric approaches [5, 6]. Indeed, to fit the lifetime distribution at the highest ages, parametric models are generally used. In the Human Mortality Database [7] for example, the age-specific pattern of mortality in an open age interval is assumed to follow the Kannisto model [8]. An alternative is the Gompertz distribution [9–11]. Parametric models for ungrouping are developed for particular applications and based on parametric assumptions for the underlying distributions, i.e. data are assumed to follow a specific trajectory. They fit specific sets of data very well and their estimation leads to few interpretable parameters; however they lack flexibility because they cannot describe a wide range of differently shaped distributions, e.g. age-at-death or age-at-onset from various diseases. We focus therefore on non-parametric methods where no assumptions about the distribution of the data are made. This allows us to ungroup age-specific patterns that follow very different trajectories and that are not described by a particular parametric family.

The paper is structured as follow: First, we briefly review different methods for ungrouping aggregated data. We then show the reasoning behind the selection of the different approaches for the comparison study and we analyze them first via a simulation study and then through an empirical data application. We conclude with a discussion and a conclusion.

Our aim here is to compare and evaluate different existing non-parametric approaches for ungrouping data with particular emphasis placed on the open-ended intervals of coarse histograms.

## Methods

### Non-parametric methods for ungrouping aggregated count data

#### Kernel density estimators

A generalization of and improvement over histograms is kernel estimation: a non-parametric method to estimate smooth probability density functions. Assume that we observe a complete sample *x*_1_,…,*x*_*n*_ drawn from an unknown continuous distribution *f*(*x*). The kernel density estimator for *f*(*x*) is $\widehat {f(x)}= \frac {1}{nh} \sum _{i=1}^{n} K (\frac {x - X_{i} }{h})$, where *K*() is the kernel function and *h* a smoothing parameter known as the bandwidth. Each kernel function is centered at each data point and the estimator smooths out the contribution of each observed data point over a local neighborhood. Here the choice of the bandwidth is crucial for the resulting estimate: If the parameter is too small, the fitted curve is undersmoothed and contains too many fluctuations; in contrast, oversmoothing occurs when the parameter is too big and most of the underlying structure is obscured. It is often difficult to choose the optimal level of smoothing: Plug-in and cross-validation are generally used in this respect. Kernel estimators are applied for example to age-specific mortality data as a graduation technique to overcome abrupt changes of crude data [12].

However, when observations are grouped in a histogram we do not know the exact locations of the data points, we only know that they lie within some intervals. In one early attempt of extending kernel density estimation for grouped data, Scott and Scheather (1985) treated all observations as if they were equal to the midpoints of their corresponding intervals. This approach performs well when the grouping scheme is fine, but produces poor results with coarse intervals [13]. A nonlinear variant of kernel density estimation has been proposed by Blower and Kelsall (2002) [6]. Here the true and unknown value of *K*(*x*−*x*_*ji*_), with *j* standing for intervals, is replaced by its expectation *E*{*K*(*x*−*x*_*ji*_)}. A smooth estimate of the underlying density *f*(*x*) is obtained through an iteration using the density of the histogram of observed counts as the initial point (see Figure 1 in Blower and Kelsall (2002) [6]). More recently, Wang and Wertelecki (2013) [14] proposed a bootstrap type kernel density estimator for binned data. Another procedure for local likelihood estimation for data aggregated into intervals, based on conditional expectation, was proposed by Braun et al. (2005) [15]. The latter two methods can cope with coarser intervals.

**Table 1:** Selected ungrouping methods for comparison

Method and references	Abbreviation	Program for estimation
Bootstrap kernel density estimator [14, 26]	bootkde	bda R package, bde function, bootkde method
Piecewise cubic Hermite interpolating polynomial [21, 27]	hermite spline	signal R package, interp1 function, pchip method
Spline interpolation with Hyman filter [5, 18, 28]	hyman spline	demography R package, cm.spline function
Iterated conditional expectation kernel density estimator [15, 29]	ickde	ICE R package, ickde function
Penalized composite link model [22]	pclm	R code in Rizzi et al. (2015)

#### Spline interpolation

A spline is a smooth numeric function that is piecewise defined by polynomial functions connected by points called knots. To interpolate data points with splines, polynomials are fitted piecewise resulting in a continuous curve that passes through each of the known data points. The degree of the polynomial is arbitrary but usually second or third orders polynomials are chosen to ensure smoothness, meaning that the spline has continuous derivatives of the first or second order.

Spline interpolation applied to age-specific data was illustrated by McNeil et al. (1977) [16] and based on Schoenberg (1964) [17]. When spline interpolation is used to estimate single-year age distributions from grouped data, the cumulative number of counts is interpolated since the only known values are those at the boundaries of each age group. If we observe data points (*x*_*i*_,*y*_*i*_) with *i*=1,..,*n*, where *x*_*i*_ correspond to the sequence of age intervals and *y*_*i*_ to the cumulative numbers of death up to age *x*_*i*_, the spline function *F*(*x*) interpolates all points and consists of polynomials between each consecutive pair of knots *x*_*i*_ and *x*_*i*+1_. Then, to obtain the death counts for each individual age group, one proceeds by differencing, i.e. *f*(*x*)=*F*^′^(*x*)=*F*(*x*+1)−*F*(*x*), where *f*(*x*) stands for the single-year age-at-death distribution. Spline interpolation is extensively used. It is applied for example by the Human Mortality Database [7] to split aggregated death counts grouped into 5-years age classes, see [8] Appendix B of the protocol. However, the method does not provide reliable estimates for open-ended age groups since the spline function starts declining at old ages leading to erroneous death counts estimates by single year of age that are negative [5]. To overcome the problem Wilmoth et al. use parametric models to fit the late life span, see [8] Appendix C of the protocol. Smith et al. (2004) [5] instead propose and apply a monotonicity constraint, the Hyman filter [18], to cubic spline interpolation for ungrouping deaths of Australian females (see Figure 1 in Smith et al. (2004) [5]). Another method that ensures non-negative values is the piecewise cubic Hermite interpolating polynomial used in the Human Fertility Database [19–21].

#### Penalized composite link model

Another method to ungroup aggregated counts is the penalized composite link model [22, 23]. It is based on the composite link model [24], which in turn extends standard generalized linear models [25]. The observed counts *y*_*i*_, with *i*=1,…,*I* number of intervals, are interpreted as realizations from Poisson distributions with expected value *μ*_*i*_. This expected value results from grouping a latent expected distribution *γ*_*j*_, with *j*=1,…,*J* number of narrower intervals, into *I* histogram bins (see Figure 1 in Rizzi et al. (2005) [22]). This latent sequence represents the true distribution that is estimated from the composite data by maximizing a penalized likelihood $L^{*} = L - \frac {\lambda }{2} P$, with *λ* the smoothing parameter and *P* a roughness penalty. The problem would be ill-defined if the likelihood would not be penalized. This results in the latent distribution to be smooth, that is neighboring elements of the estimated sequence do not differ drastically. The weight of the penalty *λ*, which controls the smoothness of the estimates, is tuned using the Akaike’s Information Criterion (AIC): The value of *λ* that gives the minimum of AIC is chosen. Eilers (2007) [23] showed that the penalized composite link model can be estimated by an appropriately modified version of the iteratively reweighted least squares (IRWLS) algorithm.

### Selection of ungrouping methods for comparison study

We retrieved from the literature methods that estimate detailed distributions from coarsely grouped data. To conduct the search we selected ‘density estimation binned data’, ‘spline interpolation demographic data’, ‘expanding abridged life table’, ‘protocol human mortality database’ as keywords in Web of Science, PubMed and Google Scholar. After screening, we obtained 36 potentially eligible studies. Moreover by searching ‘density estimation binned data statistical software’ and ‘spline interpolation in R’ we found 7 potentially relevant R packages. Among these searches we chose individual methods that are non-parametric; that always result in positive estimates, since a negative number for death counts or onset of a disease is an impossible result; and that are implemented in statistical software so that they can be readily used by health researchers. Therefore we excluded:

- 7 studies requiring additional information for input data;
- 10 parametric models;
- 6 spline interpolation methods allowing negative estimates;
- 8 studies without software implementation or temporarily disabled.

The 5 methods included in the comparison study are a bootstrap kernel density estimator (bda R packge, bde function, ‘bootkde’ method) [14, 26]; a piecewise cubic Hermite interpolating polynomial (signal R package, interp1 function, ‘pchip’ method) [27]; a spline interpolation with Hyman filter (demography R package, cm.spline function) [5, 28]; an iterated conditional expectation kernel density estimator using a local constant (ICE R package, ickde function) [15, 29]; and the penalized composite link model [22] (R code in Appendix 2 of the paper by Rizzi et al. (2005) [22]). A compact overview is given in Tstatistical softwares Stata (pchipolate function) and Matlab (pchip function). For an extensive explanation of the methods refer to [14, 15, 18, 21, 22] respectively.

### Simulation study design

We conduct a simulation study to compare the performance of the selected techniques for ungrouping age-at-death distributions. We choose as target distribution the Weibull distribution, which is a popular lifetime model used in survival analysis to describe age-specific mortality patterns [30–32]. Data are simulated from a Weibull distribution $f(x) = \frac {\alpha }{\beta } \frac {x}{\beta }^{(\alpha -1)}exp(-\frac {x}{b})^{\alpha } $ with shape *α*=8 and scale *β*=82. Parameters are arbitrarily chosen to resemble an age-at-death distribution: A shape parameter >1 indicates that mortality increases with age, characteristics of the human aging process; while the scale parameter determines the dispersion of the probability density function, thus the larger the *β* value, the more spread out the density. *X* represents the age variable that ranges from 0 to 115 by 1-year age step: This range is a reasonable support for the Weibull density since it contains 99.9999 *%* of the probability mass. Two sample sizes are considered. Thus, from the Weibull distribution 500 datasets of size *n*=200 and 500 datasets of size *n*=1000 are generated. The simulated data are then grouped according to two different schemes: One with relatively narrow intervals of equal length, i.e. 5-years age classes; the other with 5-years age classes plus a wide open-ended interval starting at age 85. For each grouped dataset an interval with 0 counts is added at the right hand tail of the distribution from age 115 up to age 130 where no observation is expected in practice. We apply the selected methods to the coarsely grouped data and for each scenario we compare the 500 estimates of each method with the true density. The scenarios of the simulation study are displayed in Table 2.

**Table 2:** Simulation study scheme

	Scenario 1	Scenario 2	Scenario 3	Scenario 4
Distribution	Weibull	Weibull	Weibull	Weibull
Sample size	*n*=200	*n*=1000	*n*=200	*n*=1000
Age groups	5-years	5-years	5-years with 85+	5-years with 85+

For each scenario 500 simulation repetitions

To study which approach performs best, we measure how close the fitted densities are to the true one that generates the data via three indicators. The integrated absolute error (IAE), i.e. $\sum |\hat {f(x)} - f(x)|$, is a straightforward measure that indicates how much the fitted density $\hat {f(x)}$ differs from the true one *f*(*x*) in absolute values; all differences are weighted equally. A performance index related to the integrated absolute error but more frequently used is the integrated squared error (ISE), i.e. $\sum (\hat {f(x)} - f(x))^{2}$. Here the differences between fitted density and corresponding true values are each squared; therefore the integrated squared error highlights the differences by giving more weight to large errors compared to the integrated absolute error. To provide a likelihood-based distance measure for densities we use the Kullback-Leibler distance [33], i.e. $\sum f(x) log [f(x) / \hat {f(x)}] $, which measures the information lost when the fitted density is used to approximate the true one. This latter index can be seen as a goodness-of-fit statistic for the model $\hat {f(x)}$, measuring the distance between actual data and the model used. All three measures have a minimum that equals 0 and therefore lower resulting values indicate a better model performance.

### Empirical application of ungrouping methods to age-specific cancer data

Free access of age-specific data by single-year of age for various causes of death or onset of diseases is not extensive and it might be of interest to estimate distributions by single-year of age from these data available in coarser age-groups. We therefore tested the models on age-specific cancer death counts and number of diagnoses. We obtained the true values of those distributions from the NORDCAN Database [34] and we analyzed age-specific all site including non-melanoma skin cancer deaths in Denmark in 2010 and age-specific incident testis cancers in Denmark in the years 1980, 1990, 2000 and 2010 combined. The death data are classified according to the International Classification of Diseases, Tenth Revision (ICD-10, codes CXX.X + D09.0-1+ D30.1-9 + D35.2-4 + D41.1-9 + D32-33 + D42-43 + D44.3-5 + D46-47) and the incident testis cancer data are also classified according to ICD-10 (code C62). All data are collected by single-year of age from age 0 up to the last age of recorded events. They serve as a ‘golden standard’ for comparison with the estimates of the different ungrouping methods. Analyzing age-at-death for total cancers and age-at-onset of testis cancer allows the study of differently shaped distributions: In the first setting there is a right-skewed distribution with a peak around age 75; in the second example the distribution increases steeply after childhood with a peak between ages 25-30 and then declines, resulting in a long right-hand tail. Registered cancer deaths in 2010 amounted to 15390, of which 2410 occurred at ages $\geqslant 85$. For the years 1980, 1990, 2000 and 2010 combined, 1020 cases of testis cancer were diagnosed. Those four years are assembled to avoid too many wiggles in the distribution, even though all methods are able to cope with that. The key message here is that we are able to study considerably different distributional shapes and sample sizes.

To compare the performance of the selected methods, we grouped the death counts into 5-year age classes first and then into 5-year age classes with an open-ended interval starting at age 85. It is the age-grouping scheme used to release data by the Danish Cancer Register and the NORDCAN Database. We apply the different models to the artificially grouped data and provide graphical representation of the different ungrouped estimates against the empirical counts. For comparison we propose the three distance measures used in the simulation study: Integrated absolute error (IAE), integrated squared error (ISE) and Kullback Leibler distance (KL).

## Results

### Simulation study results

**Fig. 1:**
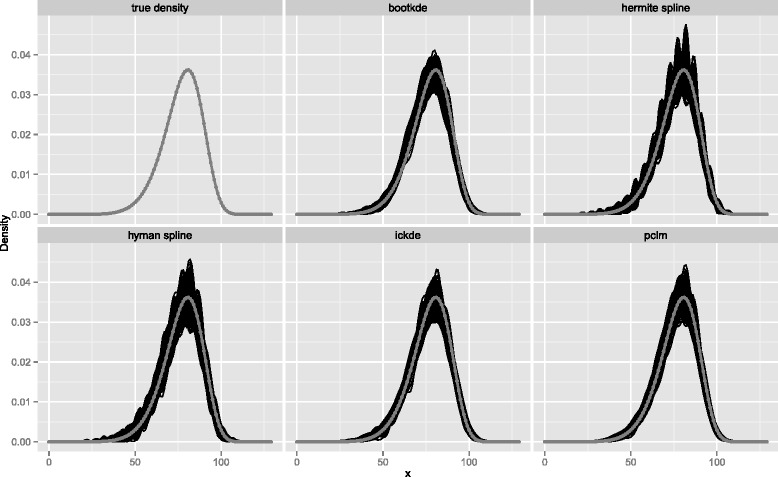
Weibull true density (*gray line with overplotted points*) and models’ fitted densities from 5-years age groups and *n*=1000 (*black lines*)

We first study the performance of the different approaches when the simulated data are artificially grouped in relatively large bins of equal size, i.e. 5-years age classes. We compare the resulting density estimates in single-year of age from 0 up to 130 years: Fig. 1 shows the target distribution and 500 estimates for each method for sample size *n*=1000. All methods capture the fit and the shape of the target distribution: The estimated Weibull densities are close to the true density and they look graphically reasonable (Fig. 1). For the kernel density estimators, the crucial choice of the optimal bandwidth is computed automatically in the bootstrap kernel density estimation (bootkde), where the procedure used is the minimization of the integrated squared error. In the iterated conditional expectation kernel density estimation (ickde), the bandwidth selection is partially left to the user. The user can choose among two procedures to obtain the optimal bandwidth suggested by the authors: A quick one implements the plug-in methodology (KernSmooth package, dpik function in R); and the other one uses likelihood cross-validation (ICE package, bickde function in R), which has the inconvenience of being very slow. In the framework of our simulation study we tried both approaches and found that the plug-in method gives considerably better results (Figs. 1 and 3, ickde panel); the likelihood cross-validation lead instead to significant oversmoothing. Therefore all reported estimates of the simulation study for the iterated conditional expectation kernel density estimation are obtained via plug-in method for bandwidth selection.

**Fig. 2:**
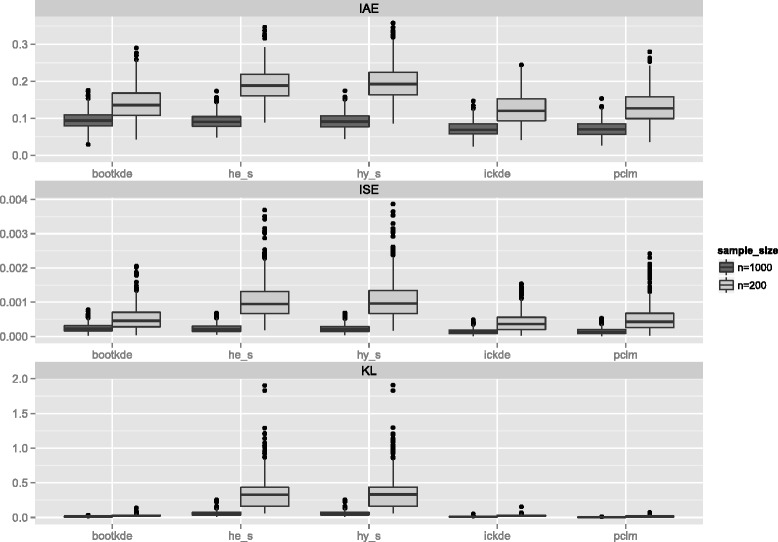
Measures of performance for Weibull density from 5-years age groups. Integrated absolute error (IAE), Integrated squared error (ISE), Kullback Leibler distance (KL)

To better analyze how far apart the target distribution and the estimates for each method are, three distance measures, integrated absolute error (IAE), integrated squared error (ISE), and Kullback Leibler distance, are presented in Fig. 2. Each boxplot shows the distance of each of the 500 estimates to the true density. The closer the distance is to 0, the better the performance. For a small sample size, the two spline interpolation methods (he_s and hy_s) show poorer results. The iterated conditional expectation kernel density estimation (ickde) and the penalized composite link model (pclm) slightly outperform the bootstrap kernel density estimator (bootkde). A bigger sample size leads to a decrease in all three measures of distance for all five methods, which show a similar performance as seen in Fig. 1.

**Fig. 3:**
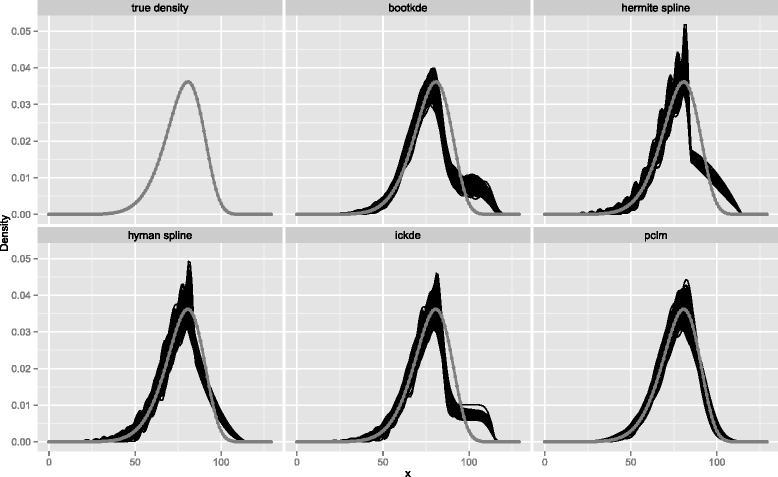
Weibull true density (*gray line with overplotted points*) and models’ fitted densities from 5-years age groups with open-ended age interval 85+ and *n*=1000 (*black lines*)

In our comparison study we put particular emphasis on the last open-ended intervals of coarse histograms, as they are commonly encountered in human age-at-death distributions. Therefore, we analyze a second grouping scheme with the simulated data aggregated into intervals of 5 years of age with a last group starting at age 85. The maximum age is set at 115 years, after which no deaths are reasonably observable and the histogram is complemented with an age group from 115 to 130 with 0 counts. Target distribution and results for the estimated densities with *n*=1000 are illustrated in Fig. 3. The penalized composite link model (pclm) is the method least affected by the wide age interval at the right-hand tail of the distribution. Both kernel density estimators (bootkde and ickde) fail in correctly distributing the information of the wide age interval in the tail area. Also, the piecewise cubic Hermite interpolating polynomial (hermite spline) has limitation in that; moreover it shows a sudden twist just before the wide interval at age 85 starts because the continuity of the second derivative is not guaranteed. The spline interpolation with Hyman filter (hyman spline) redistributes the data more smoothly, however it does not succeed in reproducing the right-hand tail of the true density with perfect accuracy.

**Fig. 4:**
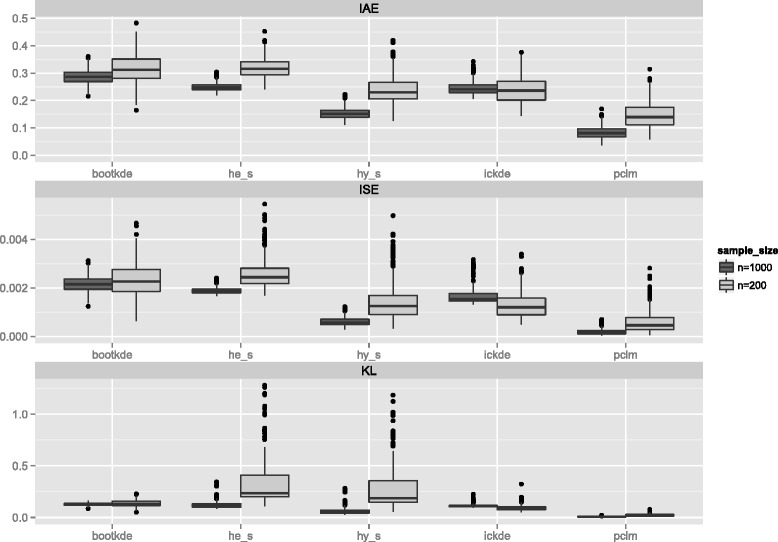
Measures of performance for Weibull density from 5-years age groups with open-ended age interval 85+. Integrated absolute error (IAE), Integrated squared error (ISE), Kullback Leibler distance (KL)

The boxplots for the distance measures in Fig. 4 confirm our findings. The penalized composite link model (pclm) clearly outperforms all other approaches for both sample sizes.

### Empirical application results

**Fig. 5:**
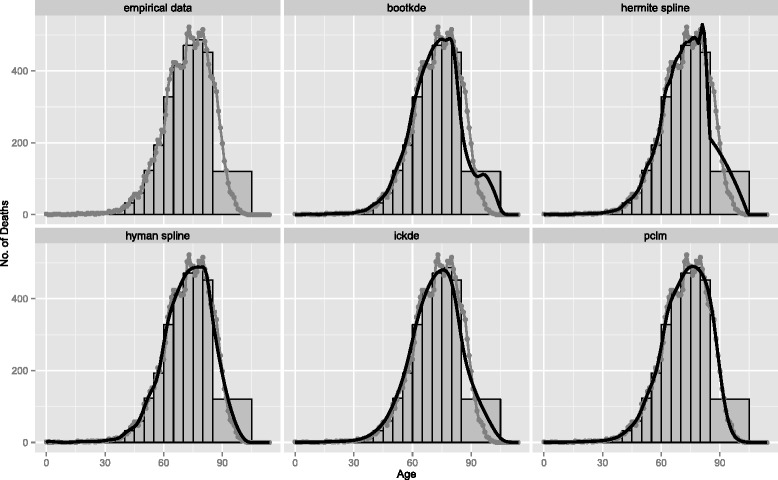
Age-at-death for all cancers in Denmark for 2010. Empirical data (*gray line with overplotted points*), grouped counts (histogram) and models’ estimates from 5-years age groups with open-ended age interval 85+ (*black smooth lines*)

We now study how the different methods for ungrouping behave in an empirical application. We start by analyzing age-specific cancer deaths in Denmark in 2010. Figure 5 reports the estimated distributions together with the empirical data for age groups of 5 years with open-ended intervals. For the results of equal interval length of 5 years we refer to the Additional file 1. Conclusions previously found in the simulation study are here confirmed, even with a sample size that increases by 15 times compared to the bigger sample size considered in the simulation study.

The last events empirically recorded correspond to age 102. We assume that age 105 is the last age at which death from cancer can be observed and we complete the histogram with an extra interval from age 105 up to age 115 with 0 counts: No cancer deaths after age 105 are expected and empirically justifiable. The added interval is essential for the two kernel density estimators (bootkde and ickde) and the penalized composite link model (pclm) to efficiently redistribute the grouped observations in the right-hand tail. These methods can therefore cope with stretches of 0 counts. The spline with Hyman filter and the Hermite polynomial, as interpolating methods, are less affected by extra information beyond the maximum age-at-death assumed. However, the added zeros also make the splines behave better at the very extreme ages when the maximum age-at-death is unknown. We noticed that the spline with Hyman filter would slightly benefit if the exact maximum age, at which the last events are observed, would be known (see Additional file 2). This however rarely occurs in practice and an assumption about the maximum age at which an observation is reasonable has to be made. In contrast with the simulation study, to optimally select the bandwidth of the iterated conditional expectation kernel density estimator (ickde) we used likelihood cross-validation (ICE package, bickde function in R). This outperformed the plug-in method (KernSmooth package, dpik function in R) which resulted in unreasonable undersmoothing.

**Fig. 6:**
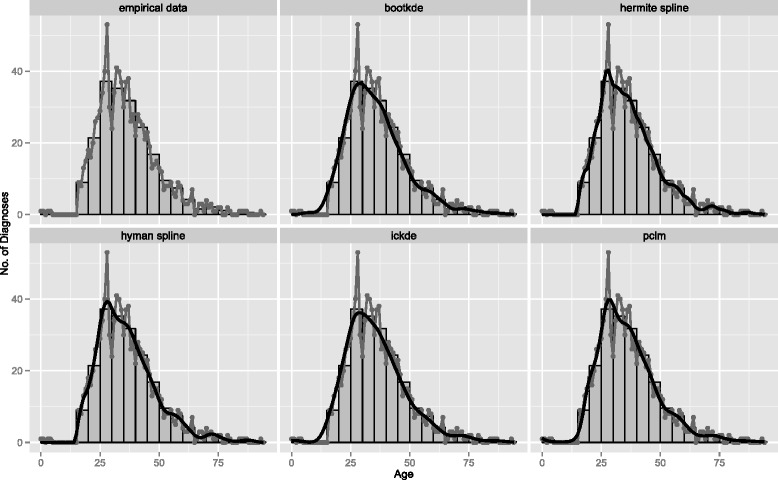
Age-at-onset of testis cancer in Denmark for 1980, 1990, 2000 and 2010 combined. Empirical data (*gray line with overplotted points*), grouped counts (histogram) and models’ estimates from 5-years age groups (*black smooth lines*)

In addition to overall age-specific cancer deaths, we apply the ungrouping methods to age-at-onset of testis cancer which is characterized by a completely different shape. Here the strength of these non-parametric methods is that they can model a wide range of distributions. This is useful in practical analysis where age-at-death from various causes of deaths or age-at-onset of different diseases follow disparate age-specific patterns, e.g. bimodal [35], skewed to the right [36] or to the left [37]. As an example we illustrate the true distribution and the estimated results for the age at diagnosis of testis cancer, prominent particularly at young adult ages, in Fig. 6. With a small sample size, empirical data are noisier compared to the previous application and all smooth estimated distributions show limitations in capturing abrupt fluctuations, in particular the one around age 30. The two kernel density estimators (bootkde and ickde) lead to very smooth distributions, while the two spline interpolation methods (hermite and hyman splines) fit some of the wiggles in the data better.

**Fig. 7:**
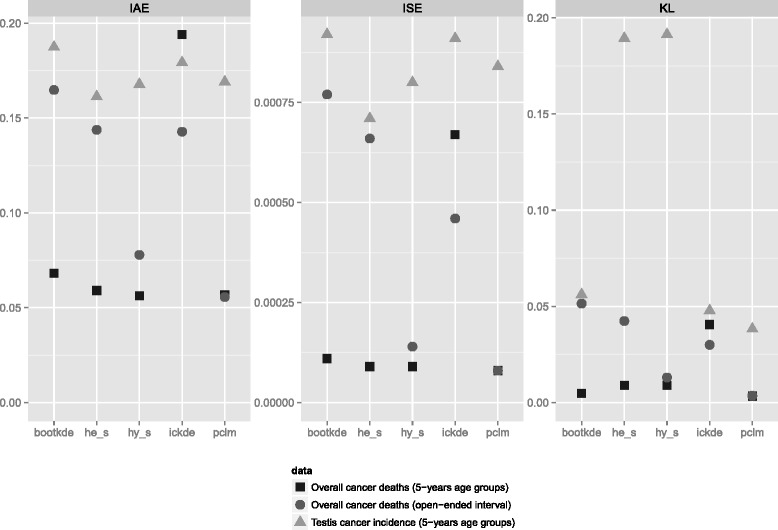
Measures of performance for the empirical data analyzed: Overall cancer deaths from 5-years age groups (*black squares*); Overall cancer deaths from 5-years age groups with open-ended age interval 85+ (*dark gray circles*); Testis cancer incidence from 5-years age groups (*light gray triangles*). Integrated absolute error (IAE), Integrated squared error (ISE), Kullback Leibler distance (KL)

To highlight the comparison between the models we report the three divergence measures for all three ungrouped estimated datasets in Fig. 7. The estimated distributions of overall cancer death from 5-years age groups show a small divergence with respect to the true empirical data: All methods are similar in this setting, except for the iterated conditional expectation kernel density estimator which oversmooths the data. The spline interpolation with Hyman filter (hy_s) and the penalized composite link model (pclm), in particular, outperform instead all competing methods in case of the open-ended interval. When empirical data are noisy and characterized by sudden fluctuations in adjacent years of age, as in the age-at-onset from testis cancer, all measures indicate higher disparity as expected.

## Discussion

We have compared different methods to split age-specific aggregated data into a fine grid of single-year ages. The selected methods for the comparison can be divided in three main groups: (i) kernel density estimators, i.e. a bootstrap kernel density estimator and an iterated conditional expectation kernel density estimation using a local constant; (ii) piecewise polynomial interpolation methods, i.e. a spline interpolation with Hyman filter and a piecewise cubic Hermite interpolating polynomial; (iii) a penalized composite link model.

For the simulated data and vital statistics analyzed we found that the penalized composite link model gives the best fit in presence of wide intervals especially, followed by the spline interpolation with Hyman filter. The penalized composite link model estimates the most likely original distribution by smoothly redistributing the grouped observations into a fine grid. Compared to the spline interpolation methods, the penalized composite link model behaves better at the extremes and does not show abrupt changes, which occur in the cubic Hermite ploynomial when the continuity of the second derivative is not guaranteed. For the penalized composite link model the optimal smoothing parameter is found by minimizing Akaike’s Information Criterion (AIC). For kernel density estimators the choice of the bandwidth, which is crucial for the resulting estimates, is more tricky, especially when left to the user.

All selected methods are ready for use, either implemented in R packages [26–29] or with code attached to the paper [22]. They can work with input data grouped in coarse intervals of unequal width; can cope with groups of 0 counts; and deal with very different sample sizes. They are non-parametric and therefore can be applied to differently shaped grouped data, such as age-specific distributions from various causes of death or age-at-onset of different diseases, where data are published in aggregated format due to privacy protection. Other relevant applications in epidemiology consist of onset of infectious diseases grouped in days or weeks; differently categorized variables from several individual studies used in meta-analyzes; measured concentrations grouped in ranges, e.g. concentration of lead in the blood [38].

When data are very noisy and show sudden fluctuations, the smoothness assumption of the methods is more questionable. All methods show limitations in this respect, as seen in the testis cancer example. However, since we know little information from grouped observations, by also assuming smoothness, we can get some useful insights about the general pattern that the data follow. In the presence of age heaping or bad quality data it can even be beneficial.

Our study is limited to the comparison of 5 ungrouping methods that are implemented in the statistical software R. We are aware that in some research fields, this software is not extensively used. However, we believe in its further spread since it is free and open source and we aim contributing to its diffusion. Other studies in the literature compare various methods for ungrouping. They focus though on parametric and non-parametric models together that estimate specific distributions, such as overall age-specific mortality [3] or age-specific fertility [4]. They also do not tackle the problem of open-ended intervals. Here, on the contrary, we aimed to test the performance of flexible non-parametric methods that are ready to be used for a broader range of applications.

## Conclusion

Efficient methods to ungroup coarse histograms are needed in several applications. In this study we compare different methods for ungrouping vital statistics. All methods show similar results for finer age groups, i.e. of 5-years age length. However the penalized composite link model is superior when tested against data aggregated in wider groups such as open age intervals.

## Additional files

Additional file 1
**Figure S1.** Age-at-death for all cancers in Denmark for 2010. Empirical data (gray line with overplotted points), grouped counts (histogram) and models’ estimates from 5-years age groups (black smooth lines). (PDF 40 kb)

Additional file 2
**Figure S2.** Age-at-death for all cancers in Denmark for 2010. Empirical data (gray line with overplotted points), grouped counts (histogram) and Hyman spline estimates from 5-years age groups with open-ended age interval 85+. Estimates by single ages up to 115 years with interval of 0 counts starting at age 105 (dark gray line) and estimates by single ages up to the last age of empirically recorded events 102 without interval of 0 counts to conclude the histogram (light blue line). (PDF 30.3 kb)

## Abbreviations

bootkde: bootstrap kernel density estimator
ickde: iterated conditional expectation kernel density estimator
pclm: penalized composite link model
Hermite spline and he_s: piecewise cubic Hermite interpolating polynomial
Hyman spline and hy_s: spline interpolation with Hyman filter
