# Supplementary material for: Comparison of non-parametric methods for ungrouping coarsely aggregated data
Source: BMC Med Res Methodol. 2016 May 23;16:59. doi: 10.1186/s12874-016-0157-8 (PMC4877978; doi:10.1186/s12874-016-0157-8)
Supplement: Additional file 1 — Figure S1. Age-at-death for all cancers in Denmark for 2010. Empirical data (gray line with overplotted points), grouped counts (histogram) and models’ estimates from 5-years age groups (black smooth lines). (PDF 40 kb) [file 12874_2016_157_MOESM1_ESM.pdf]

Additional file 1

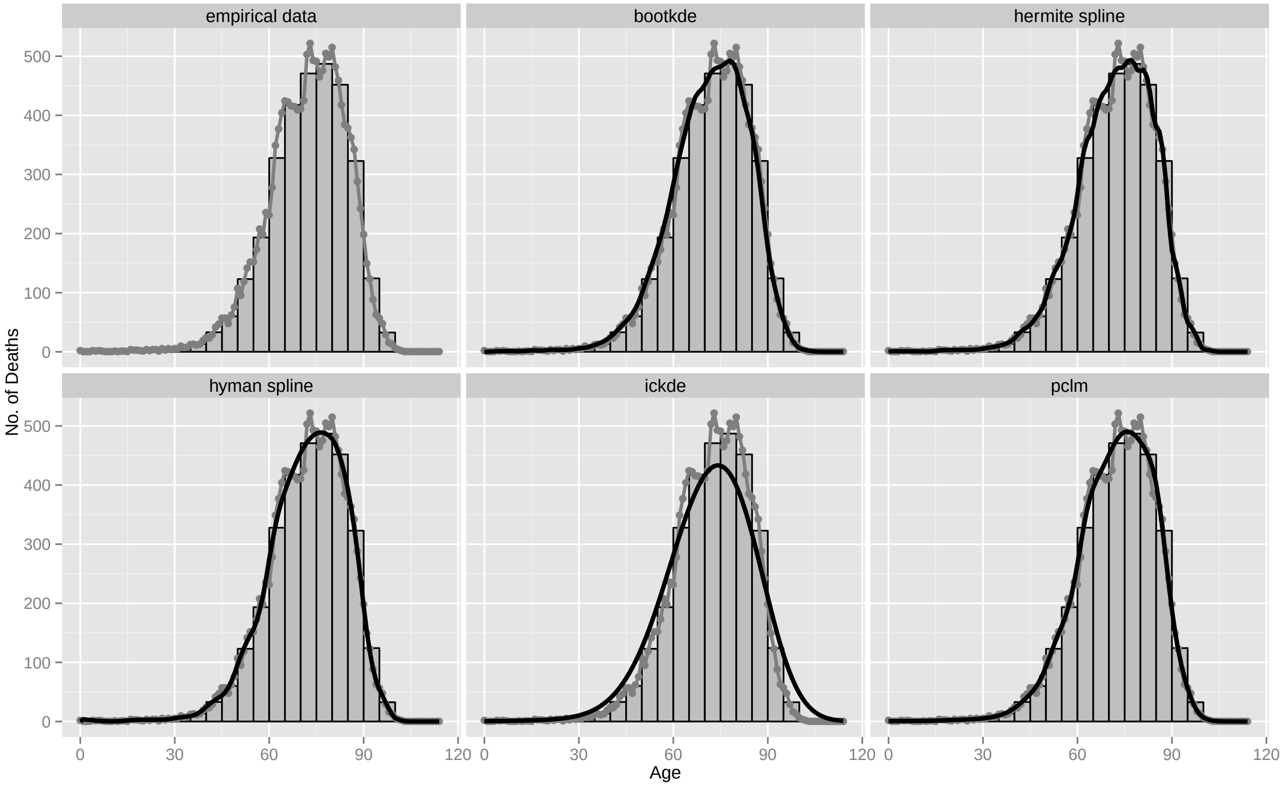

Additional Figure 1: Age-at-death for all cancers in Denmark for 2010. Empirical data (gray line with overplotted points) and models' estimates from 5-years age groups (black smooth lines).
