# Supplementary material for: Comparison of non-parametric methods for ungrouping coarsely aggregated data
Source: BMC Med Res Methodol. 2016 May 23;16:59. doi: 10.1186/s12874-016-0157-8 (PMC4877978; doi:10.1186/s12874-016-0157-8)
Supplement: Additional file 2 — Figure S2. Age-at-death for all cancers in Denmark for 2010. Empirical data (gray line with overplotted points), grouped counts (histogram) and Hyman spline estimates from 5-years age groups with open-ended age interval 85+. Estimates by single ages up to 115 years with interval of 0 counts starting at age 105 (dark gray line) and estimates by single ages up to the last age of empirically recorded events 102 without interval of 0 counts to conclude the histogram (light blue line). (PDF 30.3 kb) [file 12874_2016_157_MOESM2_ESM.pdf]

Additional file 2

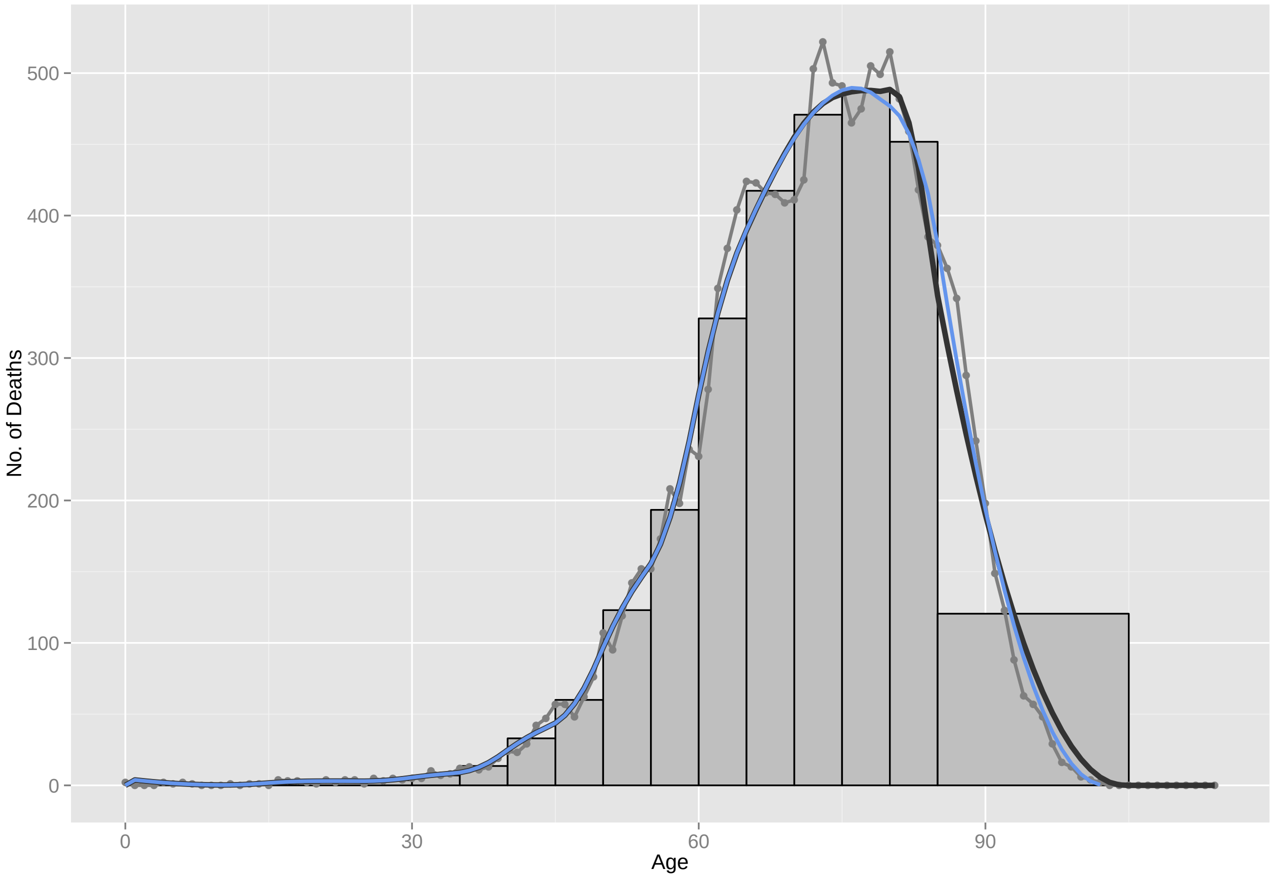

Additional Figure 2: Age-at-death for all cancers in Denmark for 2010. Empirical data (gray line with overplotted points) and Hyman spline estimates from 5-years age groups with open-ended age interval 85+. Estimates by single ages up to 115 years with interval of 0 counts starting at age 105 (dark gray line) and estimates by single ages up to the last age of empirically recorded events 102 without interval of 0 counts to conclude the histogram (light blue line).
